# Pd-catalysed [3 + 3] annelations in the stereoselective synthesis of indolizidines

**DOI:** 10.1186/1860-5397-3-8

**Published:** 2007-02-08

**Authors:** Olivier Y Provoost, Andrew J Hazelwood, Joseph P A Harrity

**Affiliations:** 1 Department of Chemistry, University of Sheffield, Brook Hill, Sheffield, S3 7HF, UK; 2 Synthetic Chemistry, GlaxoSmithKline Research and Development, Gunnels Wood Road, Stevenage, Hertfordshire, SG1 2NY, UK

## Abstract

A [3 + 3] annelation of enantiomerically pure aziridine **7** provides the functionalised piperidine **8** that can be elaborated to the indolizidine skeleton in only 4 steps with good stereocontrol.

## Introduction

**Scheme 1:**
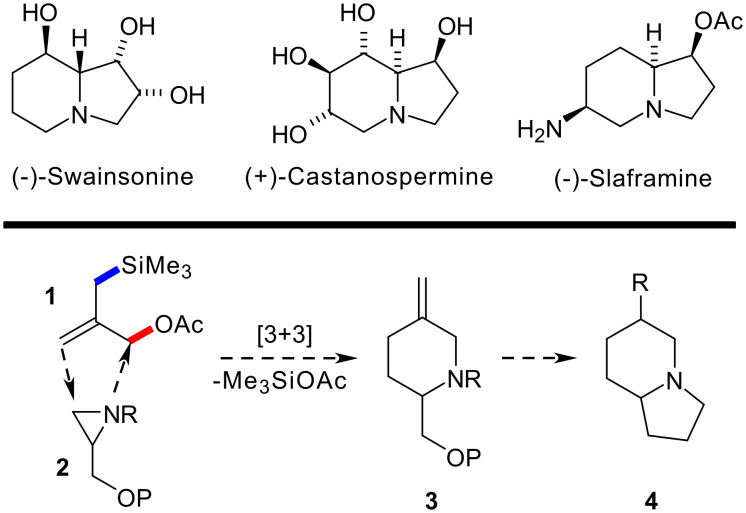
[3 + 3] Annelation approach to indolizidine skeleta.

Indolizidine alkaloids represent one of the most structurally diverse classes of natural products and have attracted considerable attention because of their varied biological activity (some examples are illustrated in Scheme 1) [1]. Recent studies in our labs have demonstrated that a range of piperidine alkaloids, [2–6] including quinolizidine based targets, [7–8] can be prepared stereoselectively through the employment of a [3 + 3] annelation strategy [9]. This approach exploits the commercially available reagent **1** developed by Trost [10] that employs a nucleophilic allylsilane motif in conjunction with an allylic acetate moiety. In an effort to expand our studies to new structural classes, we have turned our attention to the employment of this technique in the synthesis of indolizidines. Specifically, and as outlined in Scheme 1, we envisaged that a key piperidine intermediate **3** could be prepared in enantiomerically pure form and converted into a functionalised indolizidine intermediate **4** within a few steps. We wish to report herein our recent progress towards this goal.

**Scheme 2:**
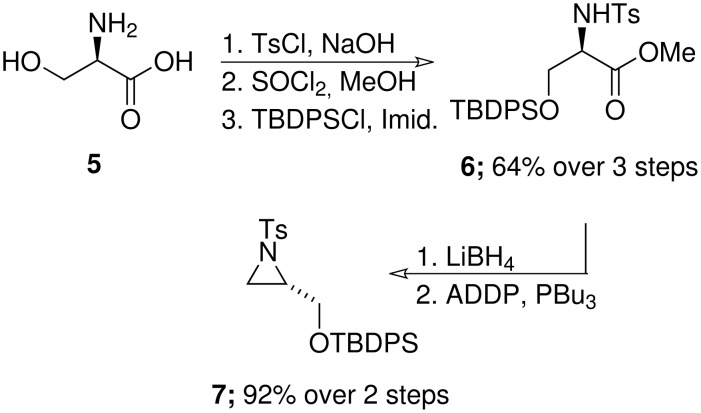
Enantiospecific aziridine synthesis (ADDP: 1,1'-(azodicarbonyl)dipiperidine).

Our studies began with the preparation of an appropriate precursor to the desired functionalised piperidine (Scheme 2). Specifically, we prepared an enantiomerically pure silyl protected aziridine **7** using a modification of the route described by Righi and co-workers [11]. Accordingly, tosyl protection of (*R*)-serine **5** followed by esterification and TBDPS-protection provided **6** in good overall yield. Ester reduction was carried out conveniently on multigram scale using LiBH_4_ to give an amino alcohol that was smoothly transformed to aziridine **7** after Mitsunobu condensation.

Having arrived at the key [3+3] annelation step, we decided to employ our standard conditions for the Pd-catalysed reaction. Indeed, we were pleased to find that the desired piperidine **8** could be furnished in high yield and that this reaction allowed 2–3 g of material to be made available at this stage (Table 1, Entry 1). Moreover, we took the opportunity to carry out a study into the role of *n*-BuLi in this process. Specifically, Trost described the use of this reagent as a reductant for the generation of low valent Pd required for generation of the intermediate TMM-reagent [12]. However, the ability of phosphite to carry out the reduction of Pd(II) to Pd(0) suggested to us that the annelation should proceed equally well in the absence of *n*-BuLi [13]. In an effort to clarify this issue we carried out a study of the [3 + 3] reaction in the absence of the alkyllithium reagent. As outlined in Table 1, Entries 2–4, the annelation was found to proceed in the absence of *n*-BuLi, however, in all cases the yield of cyclisation product was significantly lower than with catalyst generated by the organolithium reagent. Whilst the underlying reasons for this difference in catalyst activity are unclear at present, we speculate that *n*-BuLi may be responsible for the formation of hitherto uncharacterised phosphine ligands Bu_n_P(O^i^Pr)_3-n_ that promote the annelation over simple P(O^i^Pr)_3_. Indeed, analogous alkoxide substitution reactions of phosphites have been reported using Grignard reagents [14]. In addition, ^31^P NMR studies showed that the addition of 1 equivalent of ^n^BuLi to P(OPr^i^)_3_ gave a mixture of P(OPr^i^)_3_ and PBu^n^_3_ after 15 minutes (See Supporting Information File 1 for details). Interestingly however, we have found PBu^n^_3_ to be inefficient in [3 + 3] reactions as it appears to promote by-product formation [8]. Studies into the nature of the catalyst in the presence of *n*-BuLi are ongoing.

**Table 1:** Investigation of the [3 + 3] annelation reaction

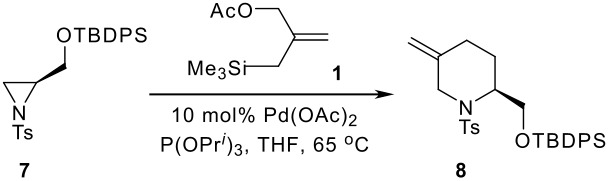			

Entry	Reductant	mol% P(O^i^Pr)_3_	Yield

1	^n^BuLi	60	74%
2	-	60	38%
3	-	40	25%
4	-	80	11%

**Scheme 3:**
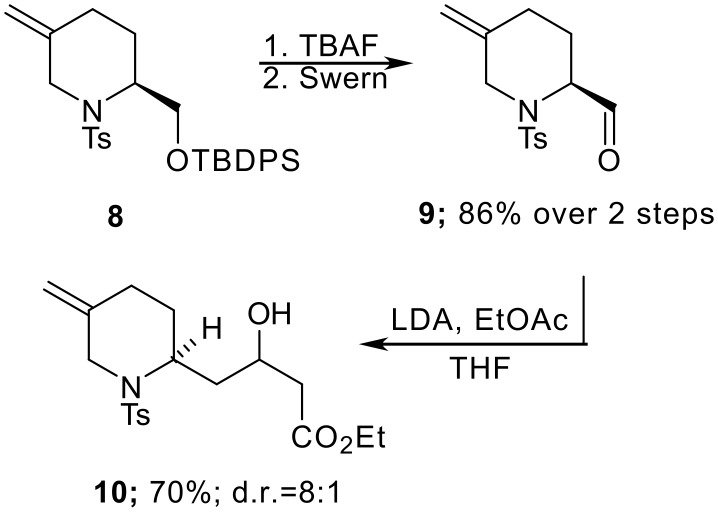
Diastereoselective aldol addition.

With the key piperidine **8** in hand, we turned our attention to the assembly of the indolizidine skeleton. Deprotection of the silyl ether proceeded smoothly and the alcohol was oxidised to the corresponding aldehyde **9** under Swern conditions. Addition of the Li-enolate of EtOAc to the crude aldehyde provided the aldol product **10** in high yield and with good diastereocontrol (Scheme 3). Notably, reduction of **9** (NaBH_4_, MeOH) followed by formation of the corresponding Mosher's ester showed a single resonance in the ^19^F NMR spectrum (235 MHz, CDCl_3_: δ-72.0) suggesting that minimal epimerisation had taken place during the oxidation process.

**Scheme 4:**
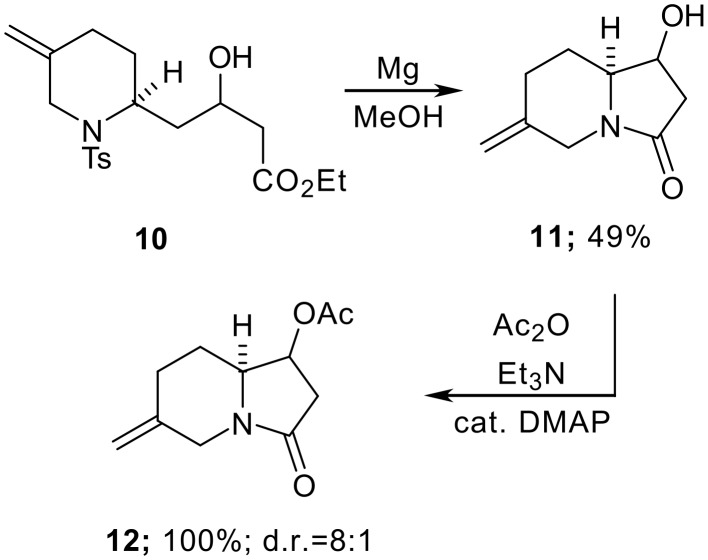
Indolizidinone formation.

We next decided to investigate the formation of the azabicycle via the deprotection of the Ts-amine moiety followed by cyclisation onto the ester. Previous work in the quinolizidine area had shown that these transformations could be achieved in one-pot by the use of Mg turnings in methanol at ambient temperature [7–8]. Indeed, subjecting **10** to these conditions provided the desired indolizidine **11**, albeit in modest yield. Finally, acetylation of the hydroxyl group provided **12** and allowed the diastereoisomers to be separated and individually characterised (Scheme 4). Unfortunately however, we were unable to determine the product stereochemistry unequivocally in either case (the ^1^H NMR data for the minor diastereomer of **12** compares well with a close analogue reported by Knapp and co-workers suggesting that the aldol addition reaction proceeds under Felkin-Anh control [see Supporting Information File 1]).

**Scheme 5:**
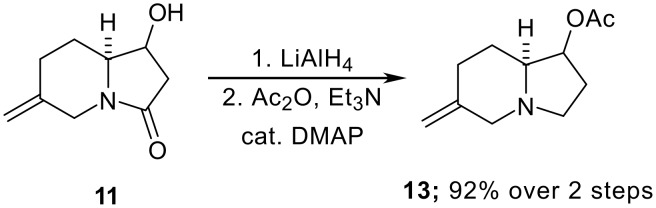
Preparation of a functionalised indolizidine.

In conclusion, we have shown that functionalised indolizidinone intermediates can be generated through the Pd-catalysed [3 + 3] annelation of aziridines and Trost's conjunctive allylsilane reagent. We have also found that reduction of the lactam unit of **11** and acetylation of the hydroxyl group takes place smoothly to provide **13**, demonstrating the potential of these intermediates for the synthesis of slaframine and related indolizidines (Scheme 5).

## Supporting Information

File 1 Supporting information. Experimental procedures and compound characterisation.

## References

[1] Takahata H, Momose T, Cordell G A (1993). The Alkaloids.

[2] Hedley S J, Moran W J, Prenzel A H G P, Price D A, Harrity J P A (2001). Synlett.

[3] Hedley S J, Moran W J, Price D A, Harrity J P A (2003). J Org Chem.

[4] Goodenough K M, Raubo P, Harrity J P A (2005). Org Lett.

[5] Provoost O Y, Hedley S J, Hazelwood A J, Harrity J P A (2006). Tetrahedron Lett.

[6] Pattenden L C, Wybrow R A J, Smith S A, Harrity J P A (2006). Org Lett.

[7] Moran W J, Goodenough K M, Raubo P, Harrity J P A (2003). Org Lett.

[8] Goodenough K M, Moran W J, Raubo P, Harrity J P A (2005). J Org Chem.

[9] Hsung R P, Kurdyumov A V, Sydorenko N (2005). Eur J Org Chem.

[10] Trost B M (1986). Angew Chem, Int Ed Engl.

[11] Righi P, Scardovi N, Marotta E, ten Holte P, Zwanenburg B (2002). Org Lett.

[12] Trost B M, Nanninga T N (1985). J Am Chem Soc.

[13] Trost B M, Renaut P (1982). J Am Chem Soc.

[14] Berlin K D, Austin T H, Stone K L (1964). J Am Chem Soc.

