# Supplementary material for: Pd-catalysed [3 + 3] annelations in the stereoselective synthesis of indolizidines
Source: Beilstein J Org Chem. 2007 Feb 8;3:8. doi: 10.1186/1860-5397-3-8 (PMC1810302; doi:10.1186/1860-5397-3-8)
Supplement: File 1 — Supporting information. Experimental procedures and compound characterisation. [file Beilstein_J_Org_Chem-03-08-s001.doc]

**Pd-Catalysed [3 + 3] Annelations in the Stereoselective Synthesis of Indolizidines**

*Olivier Y. Provoosta, Andrew J. Hazelwoodb and Joseph P.A. Harrity*a*

*aDepartment of Chemistry, University of Sheffield, Brook Hill, Sheffield,*

*S3 7HF*

*bSynthetic Chemistry, GlaxoSmithKline Research and Development, Gunnels Wood Road, Stevenage, Hertfordshire, SG1 2NY*

**Supporting Information**

##### Synthesis of (*S*)-3-Hydroxy-2-(toluene-4-sulfonylamino)-propionic acid[[1]](#footnote-2)

|  |  |  |
| --- | --- | --- |

To a solution of (*D*)-serine (8.00g, 76.1 mmol, 1 eq.) in aqueous 2M NaOH (76 ml, 152.2 mmol, 2 eq.) at 0°C was added 4-toluenesulfonyl chloride (14.5g, 76.1 mmol, 1 eq) in one portion. After 30 min, a further 10 ml 2M NaOH was added, and the reaction mixture stirred at room temperature for 24h. The reaction mixture was poured into a separating funnel, and washed with 2 portions of Et_2_O. The organic fractions were then back-extracted with aqueous 2M NaOH. The combined aqueous aqueous fractions were cooled to 0°C and acidified (pH 1) using concentrated HCl. A white precipitate formed at this point, and was collected by filtration and washed with brine, water and EtOH, and air-dried to give a white solid, 14.75 g (74%). m.p. 212-215°C (lit m.p. 214-215°C)^1^. $\left[\alpha\right]_{D}^{25}$ 11.8 (*c* 0.89, EtOH) (lit. $\left[\alpha\right]_{D}^{25}$ 13.3 (*c* 0.40, EtOH)[[2]](#footnote-3). ^1^H NMR (250MHz, (CD_3_)_2_SO) : δ2.41 (3H, s, ArC*H_3_*), 3.45-3.59 (2H, m, C*H_2_*OH), 3.70-3.81 (1H, m, HOCH_2_C*H*(NHTs)CO_2_H), 7.35 (2H, d, *J* = 8.0 Hz, Ar*H*), 7.69 (2H, d, *J* = 8.0 Hz, Ar*H*). ^13^C NMR (62.9 MHz, (CD_3_)_2_SO) : δ21.0, 58.0, 62.1, 126.6, 129.4, 138.4, 142.5, 171.3.

##### Synthesis of 3-(*tert*-Butyl-diphenyl-silanyloxy)-2-(toluene-4-sulfonylamino)-propionic acid methyl ester

|  |  |  |
| --- | --- | --- |

*N*-Tosyl-serine (4.12 g, 15.9 mmol, 1.0 eq.) in MeOH (15 ml) was cooled to 0°C. Thionyl chloride (0.812 ml, 11.1 mmol, 0.7 eq.) was added dropwise to the suspension. After the addition a drying tube was fitted and the suspension was stirred overnight at room temperature. The reaction mixture was then partitioned between Et_2_O and water, and the layers were separated. The aqueous layer was extracted with a further 2 portions of Et_2_O. The combined organic fractions were dried over MgSO_4_ and then concentrated. The crude product was dissolved in DMF (16 ml) and the solution cooled to 0°C. *tert*-butyldiphenylsilyl chloride (5.20 ml, 19.88 mmol, 1.25 eq) and imidazole (3.25 g, 47.73 mmol, 3 eq) were added. The reaction mixture was allowed to reach room temperature and stirred overnight. The reaction mixture was then partitioned between Et_2_O and water and separated. The aqueous phase was extracted with a further two portions of Et_2_O, and the combined organic fractions were washed with saturated NaHCO_3_ solution, brine and then dried over MgSO_4_. The dried organic phase was concentrated and purified by flash chromatography to provide a viscous colourless oil, 7.00 g (86%). ^1^H NMR (250 MHz, CDCl_3_) : δ0.99 (9H, s, C(C*H_3_*)_3_), 2.40 (3H, s, ArC*H_3_*), 3.53 (3H, s, OC*H_3_*), 3.80 (1H, dd, *J* = 10.0 Hz, *J* = 3.0 Hz, C*H*_2_OTBDPS), 3.95 (1H, dd, *J* = 10.0, 3.0 Hz, C*H*_2_OTBDPS), 4.03 (1H, dt, *J* = 9.0 Hz, *J* = 3.0 Hz, CH_2_C*H*(NHTs)CO_2_CH_3_), 5.49 (1H, d, *J* = 9.0 Hz, N*H*Ts), 7.25 (2H, d, *J* = 8.0 Hz, Ar*H*), 7.31-7.49 (8H, m, Ar*H*), 7.49-7.64 (2H, m, Ar*H*), 7.64-7.80 (2H, m, Ar*H*). ^13^C NMR (62.9 MHz, CDCl_3_) : δ19.2, 21.5, 26.7, 52.5, 57.5, 65.2, 127.1 (2C), 127.7, 127.8 (2C), 129.6 (4C), 132.5, 134.8 (2C), 135.5 (4C), 137.1, 143.8, 170.1. FTIR (CH_2_Cl_2_) : 3542 (br), 3285 (br), 3074 (w), 2954 (m), 2858 (m), 1747 (s), 1667 (m), 1599 (w), 1471 (m), 1428 (s), 1165 (s), 1113 (s), 821 (m), 703 (s) cm^-1^. *m/z* (TOF ES) : 434, 534 (MNa^+^, 18%). HRMS (TOF ES) calcd. for C_27_H_34_NO_5_SSi (MH^+^) : 512.1927. Found : 512.1935.

##### Synthesis of N-[2-(tert-Butyl-diphenyl-silanyloxy)-1-hydroxymethyl-ethyl]-4-methyl-benzenesulfonamide

|  |  |  |
| --- | --- | --- |

To a suspension of LiBH_4_ (0.473 g, 21.69 mmol, 3.0 eq.) in THF (15 ml) at 0°C was added ester **6** (3.70 g, 7.23 mmol, 1.0 eq.) in THF (20 ml). The reaction mixture was left warming to room temperature and stirred for 7 hours. The reaction mixture was then cooled down to 0°C and EtOAc and water were added dropwise. The aqueous layer was extracted with EtOAc, then the combined organics were washed with brine and dried over MgSO_4_. The residue was then purified by flash chromatography (5:1 Hexane:EtOAc) to give a colourless oil, 3.36 g (96%). $\left[\alpha\right]_{D}^{25}$ 2.63 (*c* 0.95, CH_2_Cl_2_). ^1^H NMR (250 MHz, CDCl_3_) : δ1.03 (9H, s, C(C*H_3_*)_3_), 1.76 (1H, br, O*H*), 2.41 (3H, s, ArC*H_3_*), 3.23-3.35 (1H, m, C*H*N), 3.48-3.60 (2H, m, C*H_2_*OH), 3.62-3.76 (2H, m, C*H_2_*OTBDPS), 5.10 (1H, d, *J* = 7.5 Hz, N*H*Ts), 7.24 (2H, d, *J* = 8.0 Hz, Ar*H*), 7.34-7.58 (10H, m, Ar*H*), 7.70 (2H, d, *J* = 8.0 Hz, Ar*H*). ^13^C NMR (62.9 MHz, CDCl_3_) : δ19.2, 21.5, 26.9, 55.8, 62.5, 63.3, 127.1 (2C), 127.8, 127.9 (2C), 129.8 (4C), 130.1, 132.5 (2C), 135.5 (4C), 137.3, 143.5. FTIR (CH_2_Cl_2_) : 3518 (br), 3279 (br), 3072 (w), 2931 (m), 2854 (m), 1597 (w), 1428 (m), 1328 (m), 1161 (s), 1113 (s), 815 (m), 703 (s) cm^-1^. *m/z* (TOF ES) : 328 (50%), 406 (100%), 484 (MH^+^, 18%). HRMS (TOF ES) calcd. for C_26_H_34_NO_4_SiS (MH^+^) : 484.1978. Found : 484.1971.

##### Synthesis of (*R*)-2-(*tert*-Butyl-diphenyl-silanoxymethyl)-1-(toluene-4-sulfonyl)-aziridine

|  |  |  |
| --- | --- | --- |

ADDP (0.877 g, 3.47 mmol, 1.2 eq) was added to a solution of amino alcohol (1.40 g, 2.89 mmol, 1 eq) and tributylphosphine (1.07 ml, 4.34 mmol, 1.5 eq) in toluene (20 ml) at 0°C and stirred for 2h at this temperature, and then at room temperature overnight. The reaction mixture was then partitioned between Et_2_O and water, and the layers were separated. The aqueous layer was extracted with a further 2 portions of Et_2_O. The combined organic fractions were dried over MgSO_4_, concentrated and then purified by flash chromatography to provide a colourless oil, 1.29 g (96%). $\left[\alpha\right]_{D}^{25}$ 31.78 (*c* 1.07, CH_2_Cl_2_). ^1^H NMR (250 MHz, CDCl_3_) : δ0.96 (9H, s, C(C*H_3_*)_3_), 2.15 (1H, d, *J* = 5.0 Hz, C*H*H aziridine), 2.40 (3H, s, ArC*H_3_*), 2.65 (1H, d, J = 11.0 Hz, CH*H* aziridine), 2.94-3.04 (1H, m, C*H* aziridine), 3.60 (1H, dd, *J* = 11.5, 5.5 Hz, C*H*HOTBDPS), 3.70 (1H, dd, *J* = 11.5, 4.0 Hz, CH*H*OTBDPS), 7.25-7.47 (8H, m, Ar*H*), 7.54-7.62 (4H, m, Ar*H*), 7.85 (2H, d, *J* = 8.0 Hz, Ar*H*). ^13^C NMR (62.9 MHz, CDCl_3_) : δ19.1, 21.6, 26.6, 30.4, 40.9, 63.1, 127.7, 128.0, 129.7, 129.8, 132.9, 135.1, 135.5, 144.4. FTIR (thin film) : 3071 (w), 2931 (m), 2858 (m), 1598 (w), 1472 (w), 1428 (m), 1164 (s), 1093 (s), 711(s) cm^-1^. *m/z* (TOF ES) : 310 (11%), 388 (100%), 466 (MH^+^, 33%). HRMS (TOF ES) calcd. for C_26_H_32_NO_3_SiS (MH^+^) : 466.1872. Found : 466.1851.

##### Synthesis of (*R*)-2-(*tert*-Butyl-diphenyl-silanoxymethyl)-5-methylene-1-(toluene-4-sulfonyl)-piperidine

|  | + |  |  |  |
| --- | --- | --- | --- | --- |

A solution of aziridine **7** (631 mg, 1.36 mol, 1.5 eq) in THF (9 ml) was treated with freshly prepared 0.14 M palladium catalyst solution[[3]](#footnote-4) (645 μl, 0.090 mmol, 0.1 eq), and 2-[(trimethylsilyl)methyl]-2-propen-1-yl acetate (192 μl, 0.90 mmol, 1.0 eq) and the reaction mixture heated at reflux for 16h. Solvent was removed *in vacuo* and the residue purified by flash chromatography (12:1 petroleumether/EtOAc) to provide a colourless oil, 346 mg (74%). $\left[\alpha\right]_{D}^{25}$ 2.03 (*c* 1.02, CH_2_Cl_2_). ^1^H NMR (250 MHz, CDCl_3_) : δ1.06 (9H, s, C(C*H_3_*)_3_), 1.34-1.52 (1H, m, C*H_2_*), 1.79-1.98 (2H, m, C*H_2_*), 2.02-2.23 (1H, m, C*H_2_*), 2.40 (3H, s, ArC*H_3_*), 3.62 (1H, d, *J* = 15.5 Hz, C*H*_2_N), 3.72-3.86 (2H, m, C*H_2_*OTBDPS), 3.96-4.08 (1H, m, C_2_*H*), 4.20 (1H, d, *J* = 15.5 Hz, C*H*_2_N), 4.66 (1H, br, C=C*H*H), 4.75 (1H, br, C=CH*H*), 7.20 (2H, d, *J* = 8.0 Hz, Ar*H*), 7.35-7.48 (6H, m, Ar*H*), 7.60-7.69 (6H, m, Ar*H*). ^13^C NMR (62.9 MHz, CDCl_3_) : δ19.2, 21.5, 24.6, 26.9, 27.1, 47.7, 53.6, 62.9, 110.3, 127.2, 127.7, 127.8, 129.4, 129.8, 133.3, 135.6, 137.7, 141.2, 142.9. FTIR (CH_2_Cl_2_) : 3072 (w), 2931 (m), 2858 (m), 1598 (w), 1428 (m), 1162 (s), 1113 (s), 703 (s) cm^-1^. *m/z* (TOF ES) : 442 (100%), 520 (MH^+^, 9%), 542 (MNa^+^, 8%). HRMS (TOF ES) calcd. for C_30_H_38_NO_3_SiS (MH^+^) : 520.2342. Found : 520.2321.

##### Synthesis of [5-Methylene-1-(toluene-4-sulfonyl)-piperidin-2-yl]-methanol

|  |  |  |
| --- | --- | --- |

A solution of piperidine **8** (257 mg, 0.494 mmol, 1.0 eq) in THF (25 ml) was cooled to 0°C and a solution of tetrabutylammonium fluoride (1 M in THF, 0.988 ml, 0.988 mmol, 2.0 eq) was added dropwise. The reaction mixture was allowed to reach room temperature and stirred overnight. The reaction mixture was then partitioned between EtOAc and water, and the layers were separated. The aqueous layer was extracted with a further 2 portions of EtOAc. The combined organic fractions were dried over MgSO_4_, concentrated and then purified by flash chromatography to provide a colourless oil, 137 mg (99%). $\left[\alpha\right]_{D}^{25}$ 60.66 (*c* 1.09, CH_2_Cl_2_). ^1^H NMR (250 MHz, CDCl_3_) : δ1.22-1.40 (1H, m, C*H_2_*), 1.43-1.59 (1H, m, C*H_2_*), 1.64-1.82 (1H, m, C*H_2_*), 2.01-2.19 (1H, m, C*H_2_*), 2.22 (3H, s, ArC*H_3_*), 2.54-2.71 (1H, br s, O*H*), 3.49-3.64 (1H, m, C*H*), 3.65-3.93 (2H, m, C*H_2_*OH), 3.72 (1H, d, *J* = 15.5 Hz, C*H_2_*N), 4.14-4.34 (1H, d, *J* = 15.5 Hz, C*H_2_*N), 4.61 (1H, br s, C=C*H*H), 4.73 (1H, br s, C=CH*H*), 7.19 (2H, d, *J* = 8.0 Hz, Ar*H*), 7.63 (2H, d, *J* = 8.0 Hz, Ar*H*). ^13^C NMR (62.9 MHz, CDCl_3_) : δ21.5, 24.8, 27.5, 47.1, 54.9, 61.8, 110.8, 127.4, 129.6, 136.9, 140.6, 143.4. FTIR (CH_2_Cl_2_) : 3388 (br), 2941 (m), 1598 (w), 1445 (w), 1157 (s), 1090 (s), 901 (w), 815 (w) cm^-1^. *m/z* (TOF ES) : 282 (MH^+^, 100%), 304 (MNa^+^, 32%). HRMS (TOF ES) calcd. for C_14_H_20_NO_3_S (MH^+^) : 282.1164. Found : 282.1175.

##### Synthesis of 5-Methylene-1-(toluene-4-sulfonyl)-piperidine-2-carbaldehyde

|  |  |  |
| --- | --- | --- |

Oxalyl chloride (1.40 ml, 16.0 mmol, 3.0 eq.) in CH_2_Cl_2_ (19 ml) was cooled to –78°C and DMSO (2.30 ml, 32.0 mmol, 6 eq.) in CH_2_Cl_2_ (38 ml) added cautiously. The resulting solution was stirred at -78°C for 15 minutes. A solution of alcohol (1.50 g, 5.33 mmol, 1 eq.) in CH_2_Cl_2_ (56 ml) was then added and the reaction mixture was stirred at –78°C for another 15 min. Et_3_N (3.00 ml, 21.3 mmol, 4 eq) was then added and the reaction mixture was allowed to reach room temperature. This was subsequently quenched with water (25 ml) and then 5% HCl (10 ml) were added, and the layer separated. The aqueous layer was further extracted with CH_2_Cl_2_ (3x) and the combined organics were dried (MgSO_4_) and concentrated. The residue was purified by flash chromatography (70:30 Ether:Hexane) to yield 90 mg (86%) as a colourless oil. ^1^H NMR (250 MHz, CDCl_3_) : δ1.37-1.58 (1H, m, C*H_2_*), 1.91-2.14 (3H, m, C*H_2_*), 2.36 (3H, s, ArC*H_3_*), 3.59-3.79 (1H, d, *J* = 14.0 Hz, C*H*_2_N), 3.85-4.00 (1H, d, *J* = 14 Hz, C*H*_2_N), 4.03-4.19 (1H, m, C*H*), 4.69 (1H, br s, C=C*H*H), 4.78 (1H, br s, C=CH*H*), 7.24 (2H, d, *J* = 8.0 Hz, Ar*H*), 7.61 (2H, d, *J* = 8.0 Hz, Ar*H*), 9.57 (1H, s, C*H*O). ^13^C NMR (62.9 MHz, CDCl_3_) : δ21.5, 24.4, 28.6, 50.0, 62.0, 112.1, 127.6, 129.8, 135.6, 139.2, 143.9, 199.7. FTIR (CH_2_Cl_2_) : 2928 (w), 2848 (w), 1731 (s), 1598 (w), 1443 (m), 1160 (s), 1097 (s), 1018 (m), 994 (m), 951 (w), 919 (w), 859 (w), 815 (m) cm^-1^. *m/z* (TOF ES) : 280 (MH^+^, 100%), 302 (MNa^+^, 20%). HRMS (TOF ES) calcd. for C_14_H_18_NO_3_S (MH^+^) : 280.1007. Found : 280.1016.

##### Synthesis of ethyl 3-hydroxy-3-(5-methylene-1-tosylpiperidin-2-yl)propanoate

|  |  |  |
| --- | --- | --- |

To a stirred solution of diisopropylamine (27 μl, 0.19 mmol, 1.6 eq) in THF (1.0 ml) at 0°C was added BuLi (73 μl, 0.18 mmol, 1.5 eq). The resulting solution was stirred for 30 minutes and cooled to -78°C before ethyl acetate (15 μl, 0.16 mmol, 1.3 eq) was added dropwise. After 30 minutes, a solution of aldehyde **9** (34 mg, 0.122 mmol, 1.0 eq) in THF (1.0 ml) was added and the reaction mixture was stirred at -78°C for an additional 30 minutes. Saturated aqueous ammonium chloride and Et_2_O were added. The separated aqueous phase was extracted with Et_2_O and the combined organic extracts were dried over MgSO_4_, filtered and concentrated. The desired compound was then obtained as an 8:1 mixture of diastereomers after flash chromatography (31 mg, 70%). ^1^H NMR (250 MHz, CDCl_3_) : δ0.96-1.14 (1H, m, C*H_2_*), 1.22 (3H, t, *J* = 7.0 Hz, major C*H_3_*), 1.20 (3H, t, *J* = 7.0 Hz, minor C*H_3_*), 1.78-1.88 (1H, m, C*H_2_*), 1.89-2.01 (1H, m, C*H_2_*), 2.19-2.31 (1H, m, C*H_2_*), 2.39 (3H, s, ArC*H_3_*), 2.53 (1H, dd, *J* = 17.0 Hz, *J* = 9.0 Hz, major C*H*_2_-C(O)), 2.56 (1H, d, *J* = 2.0 Hz, minor C*H*_2_-C(O)), 2.88 (1H, dd, *J* = 17.0 Hz, *J* = 3.0 Hz, C*H*_2_-C(O)), 3.41-3.53 (1H, br s, O*H*), 3.65 (1H, d, *J* = 16.0 Hz, major C*H_2_*N), 3.70-3.80 (1H, m, C*H*N), 4.11 (2H, q, *J* = 7.0 Hz, minor C*H_2_*CH_3_), 4.12 (2H, q, *J* = 7.0 Hz, major C*H_2_*-CH_3_), 4.24-4.37 (2H, m, C*H*OH, C*H_2_*N), 4.62 (1H, s, C=CH*H*), 4.73 (1H, s, C=CH*H*), 7.23 (2H, d, *J* = 8.0 Hz, Ar*H*), 7.66 (2H, d, *J* = 8.0 Hz, Ar*H*). ^13^C NMR (62.9 MHz, CDCl_3_) Major diasteromer only: δ14.1, 21.5, 23.1, 27.0, 38.2, 48.0, 56.0, 60.9, 65.7, 110.7, 127.4, 129.5, 137.3, 140.4, 143.3, 173.6. FTIR (CH_2_Cl_2_) : 3518 (br m), 3072 (w), 2944 (m), 1732 (w), 1655 (w), 1598 (w), 1495 (w), 1446 (m), 1227 (w), 1158 (s), 1105 (m), 1052 (m), 1018 (w), 976 (m), 902 (m), 816 (m), 708 (w) cm^-1^. *m/z* (TOF ES) : 390 (MNa^+^, 100%). HRMS (TOF ES) calcd. for C_18_H_25_NO_5_NaS (MNa^+^) : 390.1351. Found : 390.1359.

##### Synthesis of 1-hydroxy-6-methylene-hexahydroindolizin-3(5H)-one

|  |  |  |
| --- | --- | --- |

Grounded Mg turnings (1.31 g, 53.8 mmol, 30 eq) were placed in a 2-neck flask equipped with a condenser. To this was added MeOH (15 ml) and then piperidine **10** (659 mg, 1.79 mmol, 1.0 eq) in MeOH (30 ml). The resulting suspension was left stirring overnight at room temperature. HCl 1M was added to the solution and the resulting aqueous phases were extracted with EtOAc. The organics layers were combined and dried over MgSO_4_. The crude residue was then purified using flash chromatography to provide **11** as a colourless oil, 147 mg (49% yield). ^1^H NMR (250 MHz, CDCl_3_) : δ1.06-1.25 (1H, m, C*H_2_*), 2.02-2.17 (2H, m, C*H*_2_), 2.18-2.30 (1H, m, C*H_2_*), 2.30-2.45 (1H, m, C*H*_2_), 2.62 (1H, dd, *J* = 17.5 Hz, *J* = 8.0 Hz, C*H*_2_), 3.28 (1H, d, *J* = 14.5 Hz, major C*H*_2_N), 3.37 (1H, dt, *J* = 12.0 Hz, *J* = 3.5 Hz, major C*H*N), 3.45-3.55 (2H, m, minor C*H*-OH, minor C*H*_2_N), 3.95-4.04 (1H, m, C*H*OH), 4.37 (1H, d, *J* = 15.0 Hz, minor C*H_2_*N), 4.40 (1H, d, J = 14.5 Hz, major C*H_2_*N), 4.73 (1H, br, C=C*H*H), 4.81 (1H, br, C=CH*H*). ^13^C NMR (62.9 MHz, CDCl_3_) Major diasteromer only: δ31.1, 31.8, 40.4, 45.9, 65.1, 69.9, 111.3, 140.3, 171.3. FTIR (CH_2_Cl_2_) : 3370 (br), 2941 (m), 1667 (s), 1460(m), 1438 (m), 1257 (m), 1173 (w), 1086 (w), 1048 (w), 1012 (w), 906 (w) cm^-1^. *m/z* (EI) : 67 (30%), 94 (28%), 96 (75%), 167 (MH^+^, 100%). HRMS (EI) calcd. for C_9_H_14_NO_2_O : 167.0946. Found : 167.0941.

##### Synthesis of 6-methylene-3-oxo-octahydroindolizin-1-yl acetate

|  |  | 8:1 mixture |
| --- | --- | --- |

To a solution of indolizidinone **11** (22 mg, 0.13 mmol, 1.0 eq) in DCM (3 ml) at room temperature was added acetic anhydride (24 μl, 0.26 mmol, 2.0 eq), Et_3_N (37 μl, 0.26 mmol, 2.0 eq) and DMAP (catalytic amount). The resulting reaction mixture was stirred for 2h. EtOAc and water were added and the aqueous layers were extracted with EtOAc. The organics layers were washed with water and brine. The crude residue was then purified using flash chromatography to provide **12** as a colourless oil (28 mg, 100%). Careful chromatography allowed small samples of each diastereomer to be characterised spectroscopically.

Major diastereomer (tentative assignment above): ^1^H NMR (250 MHz, CDCl_3_) : δ1.17-1.38 (1H, m, C*H_2_*), 2.06 (3H, s, C(O)-C*H_3_*), 2.07-2.14 (1H, m, C*H_2_*), 2.13-2.17 (1H, m, C*H_2_*), 2.18-2.29 (2H, m, C*H_2_*), 2.80 (1H, dd, *J* = 18.0 Hz*, J* = 8.0 Hz, C*H_2_*), 3.34 (1H, d, *J* = 13.0 Hz, C*H*_2_N), 3.51 (1H, dt, *J* = 12.0 Hz, *J* = 3.0 Hz, C*H*-N), 4.53 (1H, d, *J* = 13.0 Hz, C*H*_2_N), 4.79 (1H, d, *J* = 1.5 Hz, C=C*H*H), 4.88 (1H, d, *J* = 1.5 Hz, C=CH*H*), 4.89-4.94 (1H, m, C*H*-O). ^13^C NMR (62.9 MHz, CDCl_3_) : δ21.0, 31.0, 32.0, 37.1, 45.9, 63.0, 71.4, 111.5, 140.0, 169.8, 170.6. FTIR (CH_2_Cl_2_) : 2941 (m), 2852 (w), 1739 (s), 1695 (s), 1457 (m), 1433 (m), 1237 (s), 1035 (m), 993 (w), 941 (w), 908 (w) cm^-1^. *m/z* (TOF ES) : 210 (MH^+^, 100%), 232 (MNa^+^, 80%). HRMS (TOF ES) calcd. for C_11_H_16_NO_3_ (MH^+^) : 210.1130. Found : 210.1123.

Minor diastereomer (tentative assignment above): ^1^H NMR (250 MHz, CDCl_3_) : δ1.29-1.55 (1H, m, C*H_2_*), 1.59-1.77 (1H, m, C*H_2_*), 2.01 (3H, s, C(O)-C*H_3_*), 2.05-2.22 (1H, m, C*H_2_*), 2.28-2.53 (2H, m, C*H_2_*), 2.69 (1H, ddd, *J* = 2.0 Hz, *J* = 7.0 Hz, *J* = 18.0 Hz, C*H_2_*), 3.29 (1H, d, *J* = 14.5 Hz, C*H_2_*N), 3.56-3.74 (1H, m, C*H*-N), 4.46 (1H, d, *J* = 14.5 Hz, N-C*H_2_*), 4.79 (1H, d, *J* = 1.0 Hz, C=C*H*H), 4.86 (1H, d, *J* = 1.5 Hz, C=CH*H*), 5.33-5.42 (1H, m, C*H*-O). ^13^C NMR (62.9 MHz, CDCl_3_) : δ26.1, 31.2, 33.1, 37.9, 46.1, 59.6, 71.4, 112.6, 141.0, 171.4, 173.6. FTIR (CH_2_Cl_2_) : 2929 (w), 2854 (w), 1740 (s), 1694 (s), 1434 (m), 1374 (w), 1304 (w), 1237 (s), 1179 (w), 1067 (w), 1028 (w), 941 (w), 908 (w) cm^-1^. *m/z* (EI) : 67 (40%), 96 (80%), 120 (32%), 149 (100%), 209 (M^+^, 40%). HRMS (EI) calcd. for C_11_H_15_NO_3_ (M^+^) : 209.1052. Found : 209.1059.

##### Synthesis of 6-methylene-octahydroindolizin-1-ol

|  |  |  |
| --- | --- | --- |

To a suspension of LiAlH_4_ (27 mg, 0.72 mmol, 4.0 eq.) in THF (2 ml) at 0°C was added **11** (30 mg, 0.18 mmol, 1.0 eq.) in THF (2 ml). The reaction mixture was left warming to room temperature and stirred for 4 hours. The reaction mixture was then cooled down to 0°C and water and NaOH 15% were added dropwise. MgSO_4_ was added to the resulting solution which was then filtered and concentrated. The crude residue was used directly in the next step.

##### Synthesis of 6-methylene-octahydroindolizin-1-yl acetate

|  |  |  |
| --- | --- | --- |

To 6-methylene-octahydroindolizin-1-ol (assumed 0.18 mmol, 1.0 eq) in DCM (6 ml) at room temperature was added acetic anhydride (34 μl, 0.36 mmol, 2.0 eq), Et_3_N (50 μl, 0.36 mmol, 2.0 eq) and DMAP (catalytic amount). The resulting reaction mixture was stirred for 2h. EtOAc and water were added and the aqueous layers were extracted with EtOAc. The organics layers were washed with water and brine. The crude residue was then purified using flash chromatography to provide **13** as a colourless oil (26 mg, 92%). ^1^H NMR (250 MHz, CDCl_3_) : δ1.14-1.44 (1H, m, C*H_2_*), 1.54-1.68 (1H, m, C*H_2_*), 2.03 (3H, s, minor C(O)-C*H_3_*), 2.04 (3H, s, major C(O)-C*H_3_*), 1.90-2.14 (1H, m, C*H_2_*), 2.16-2.30 (2H, m, C*H_2_*), 2.31-2.46 (3H, m, C*H_2_*), 2.58 (1H, d, *J* = 11.5 Hz, minor N-C*H*_2_), 2.80 (1H, d, *J* = 11.5 Hz, major N-C*H*_2_), 2.93-3.05 (1H, m, major N-C*H*_2_), 3.11-3.22 (1H, m, minor N-C*H*_2_), 3.44 (1H, d, *J* = 12.0 Hz, major C*H*-N), 3.57 (1H, d, *J* = 12.0 Hz, minor C*H*-N), 4.81 (2H, s, C=C*H_2_*), 4.66-4.84 (1H, m, major C*H*-O), 5.15-5.25 (1H, m, minor C*H*-O). ^13^C NMR (62.9 MHz, CDCl_3_) : δ21.1, 28.8, 29.7, 30.1, 32.3, 51.8, 58.8, 67.5, 110.4, 142.3, 174.8. FTIR (CH_2_Cl_2_) : 2970 (m), 2929 (m), 2854 (w), 1736 (s), 1654 (w), 1444 (m), 1374 (s), 1242 (s), 1157 (w), 1112 (w), 1047 (s) cm^-1^. *m/z* (TOF ES) : 196 (MH^+^, 100%), 237 (80%), 267 (82%), 319 (85%), 422 (20%). HRMS (TOF ES) calcd. for C_11_H_18_NO_2_ (MH^+^) : 196.1338. Found : 196.1336.

*(ppm)*

*-38*

*-36*

*-34*

*-32*

*-30*

*-28*

*-26*

*-24*

*-22*

*-20*

*-18*

*-16*

*-14*

*-12*

*-10*

*-8*

*-6*

*-4*

*-2*

**P(O^i^Pr)_3_**

*(ppm)*

*-38*

*-36*

*-34*

*-32*

*-30*

*-28*

*-26*

*-24*

*-22*

*-20*

*-18*

*-16*

*-14*

*-12*

*-10*

*-8*

*-6*

*-4*

*-2*

**P(O^i^Pr)_3_ + BuLi**

*(ppm)*

*-38*

*-36*

*-34*

*-32*

*-30*

*-28*

*-26*

*-24*

*-22*

*-20*

*-18*

*-16*

*-14*

*-12*

*-10*

*-8*

*-6*

*-4*

*-2*

**[P(O^i^Pr)_3_ + BuLi] + PBu_3_**

# Tentative assignment of aldol stereochemistry

1. Berry, M. B. ; Craig, D. *Synlett*, **1992**, 41. [↑](#footnote-ref-2)
2. Stoll, A. ; Petrzilka, T. *Helv. Chim. Acta*, **1952**, *35*, 589. [↑](#footnote-ref-3)
3. **Representative procedure for 0.14 M Pd catalyst used in cycloaddition reactions**

   To a suspension of Pd(OAc)_2_ (50 mg, 0.22 mmol, 1 eq.) in THF (1.61 ml) was added P(O*^i^*Pr)_3_ (0.33 ml, 1.34 mmol, 6 eq.), and then *n*-butyllithium (2.5 M in hexane, 0.18 ml, 0.44 mmol, 2 eq) was added and the resultant solution was stirred for 15 min before use. [↑](#footnote-ref-4)
